# RANO 2.0: critical updates and practical considerations for radiological assessment in neuro-oncology

**DOI:** 10.1007/s11604-025-01821-6

**Published:** 2025-06-30

**Authors:** Akihiko Sakata, Yasutaka Fushimi, Sonoko Oshima, Megumi Uto, Yohei Mineharu, Satoshi Nakajima, Sachi Okuchi, Takayuki Yamamoto, Sayo Otani, Satoshi Ikeda, Shigeki Takada, Takashi Mizowaki, Yoshiki Arakawa, Yuji Nakamoto

**Affiliations:** 1 Department of Diagnostic Imaging and Nuclear Medicine, Graduate School of Medicine, Kyoto University, 54 Shogoin Kawahara-cho, Sakyo-ku, Kyoto, 606-8507 Japan; 2 Department of Diagnostic Radiology, Shiga General Hospital, 5-4-30, Moriyama-Cho, Moriyama, 524-8524 Japan; 3 Department of Radiation Oncology, Graduate School of Medicine, Kyoto University, Kyoto, 606-8507 Japan; 4 Department of Neurosurgery, Graduate School of Medicine, Kyoto University, Kyoto, 606-8507 Japan

**Keywords:** Glioma, MRI, Treatment response criteria, RANO 2.0, Pseudoprogression, Pseudoresponse

## Abstract

Appropriate response assessment criteria are crucial for accurate evaluation of clinical trial outcomes, and numerous criteria have been proposed to address this need. With the introduction of Response Assessment in Neuro-Oncology (RANO) criteria version 2.0 (RANO 2.0) in 2023, response assessment in gliomas has evolved significantly, requiring both clinicians and radiologists to develop a comprehensive understanding of its modifications and implementation. This review first provides an overview of standard management and imaging schedule in glioma treatment. We then review the basic framework of RANO 2.0, inherited from previous response criteria, with particular emphasis on major modifications to this framework: the implementation of the Brain Tumor Imaging Protocol and the adoption of post-radiation scan as the baseline scan. Additionally, we analyze critical changes in response evaluation and interpretation, specifically focusing on the role of preliminary progressive disease assessment with confirmation scans, and the elimination of T2/FLAIR lesion measurements from enhancing tumor assessment. Through illustrative clinical cases, we demonstrate the practical application of these modifications and discuss the implementation of three distinct imaging-based categories: enhancing tumor, non-enhancing tumor, and tumors with both enhancing and non-enhancing components (in short, mixed tumor). This comprehensive narrative review provides clinicians with practical guidance for implementing RANO 2.0 in their clinical practice.

## Introduction

The Response Assessment in Neuro-Oncology (RANO) criteria version 2.0 (RANO 2.0), published in 2023, were designed as comprehensive framework for treatment response assessment in clinical trials across all glioma subtypes [1]. Here, “all” encompasses a wide range of clinical and pathological contexts, including: (1) contrast enhancement pattern, (2) newly diagnosed setting versus recurrent disease, (3) presence or absence of residual disease following initial treatment, (4) type of therapeutic intervention, (5) histological grade, and (6) molecular classification (e.g., isocitrate dehydrogenase (*IDH)* mutation status). This breadth represents an ambitious undertaking. Rather than applying a uniform assessment strategy across heterogeneous disease presentations, the effective use of RANO 2.0 requires careful consideration of these factors to appropriately identify and categorize target lesions [2].

This review begins by introducing the standard diagnostic approaches, therapeutic strategies, and imaging protocols and schedules essential for glioma management. Building upon foundational elements of previous response criteria, we explore RANO 2.0’s key modifications, particularly focusing on the standardization of imaging acquisition protocols and the implementation of post-radiation baseline scans which allows for more accurate response assessment following initial treatment. The review also discusses critical methodological changes, including the introduction of preliminary progressive disease (PD) assessment with confirmation scans and the refinement of T2/FLAIR lesion evaluation criteria. Finally, through carefully selected clinical scenarios, we show the practical implementation of these modifications across various tumor types, providing concrete guidance for response assessment in clinical practice. This work aims to bridge the gap between updated evaluation criteria and their application in everyday clinical settings.

## Standard management and imaging protocols in glioma

Glioma management requires a multifaceted approach that integrates histological grade and tumor subtype with other critical factors such as tumor location, molecular markers, and patient characteristics [3]. While maximal safe resection is a universal goal to reduce tumor burden, the feasibility of surgical intervention varies considerably based on tumor location and patient-specific factors, sometimes limiting procedures to partial resection or biopsy [4, 5]. Even after achieving gross total resection, adjuvant therapy remains necessary due to glioma infiltration beyond radiographically visible margins [6, 7]. For high-grade gliomas, after resection, the standard of care consists of the Stupp regimen, comprising concurrent temozolomide (TMZ) and radiotherapy followed by adjuvant TMZ chemotherapy [8, 9]. Tumor-treating fields are increasingly being incorporated into this therapeutic protocol to further improve outcomes [10, 11]. For low-grade gliomas, management decisions are more nuanced, with varying approaches to the timing and implementation of postoperative chemotherapy and radiotherapy based on molecular profiles and progression risk factors [12]. In recurrent disease settings, therapeutic options include repeat surgical intervention when feasible, alternative chemotherapy regimens, bevacizumab in selected cases, and consideration for radiation therapy if not previously administered or if re-irradiation is deemed appropriate [13].

In recent years, molecular biological characteristics, particularly *IDH* mutation status, have gained increasing significance in glioma classification [14–16]. While the fundamental treatment strategy remains largely unchanged, treatment selection for low-grade gliomas has changed based on molecular biological characteristics. A notable example involves *IDH*-mutant low-grade gliomas. *IDH* inhibitors have demonstrated improvement of progression-free survival (PFS) and delaying the time to the next intervention, and are expected to be incorporated into routine clinical practice [17]. Another example is a group termed molecular glioblastoma. A subset of *IDH*-wildtype gliomas that are histologically classified as lower-grade are now recognized as molecular glioblastoma [18–23]. Although definitive evidence is still lacking [24], these cases are increasingly being treated more aggressively, following protocols similar to those for glioblastoma.

**Fig. 1:**
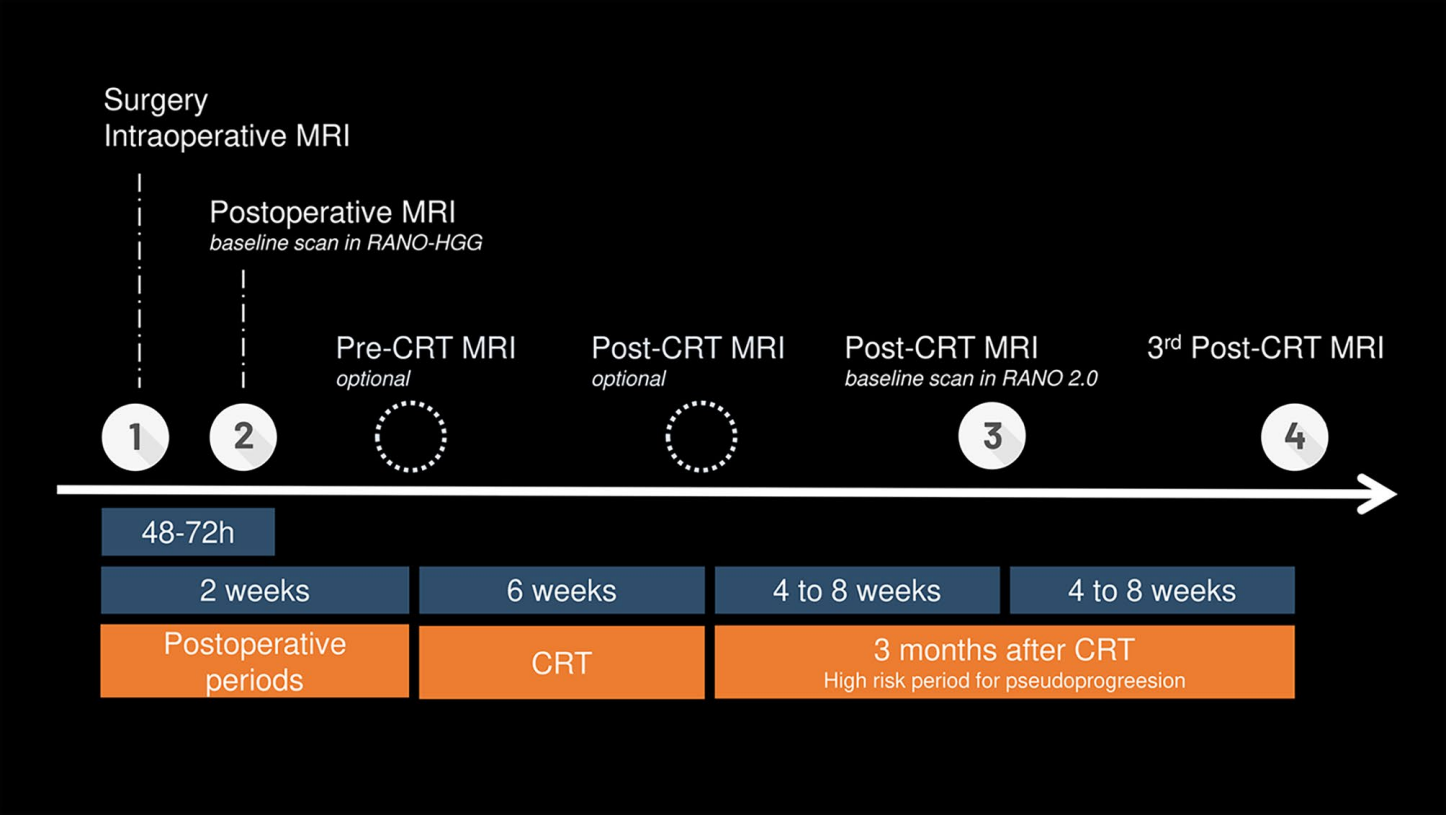
Representative treatment and imaging schedule for high-grade gliomas in Kyoto University hospital

MRI with contrast agent has become the de facto standard for appropriate assessment of treatment response. However, to our knowledge, there are no definitive guidelines regarding imaging schedules [25–27]. While several groups have conducted excellent retrospective studies [28–30], current imaging protocols for glioma appear to be based more on pragmatic considerations rather than robust evidence. Figure 1 illustrates our institution’s treatment and MRI imaging schedule for high-grade gliomas. At our institution, the extent of resection is initially verified using intraoperative MRI. The first postoperative MRI with contrast agent is performed within 48 h following surgical resection. Additional MRI examinations before initiating concurrent chemoradiotherapy (CRT) at approximately 2 weeks post-surgery and following completion of the standard 6-week radiotherapy regimen, while optional, provide valuable clinical information for treatment response evaluation. Subsequently, follow-up MRI examinations are scheduled at regular intervals of one to two months to monitor disease progression and therapeutic efficacy. This represents an observational clinical case rather than a formal clinical trial; therefore, the follow-up interval will be incrementally extended contingent upon the absence of disease recurrence. If there is a suspicion of recurrence, a further examination may be carried out after approximately one month. In low-grade gliomas, while MRI scanning intervals tend to be longer compared to high-grade gliomas, the nature of the disease necessitates long-term follow-up. Furthermore, as contrast-enhancing lesions in these cases often emerge after extended periods of post-treatment surveillance, determining how long to continue administering gadolinium-based contrast agents throughout this prolonged monitoring presents a particularly challenging clinical consideration.

Clinical trials in glioma encompass both newly diagnosed and recurrent cases. While many of these trials evaluate survival improvement through novel therapeutic agents as their primary outcome, some aim to optimize existing treatment modalities, such as radiation dose reduction in elderly patients [31, 32]. In these clinical trials, while some protocols strictly specify MRI acquisition sequences, others may accept standard institutional imaging sequences provided they meet basic requirements. The timing of imaging acquisitions is generally specified in the protocol schedules. Treatment response is evaluated by trial physicians using standardized criteria. Although response assessments are typically performed at the treating institutions, central radiologic review may be required in many trials. Discrepancies between local and central assessments can occur [33, 34], potentially affecting trial outcomes and patient management decisions. Hence, these response assessment criteria are generally required to demonstrate high inter-observer reproducibility regardless of the evaluating physicians, and to show strong correlation with patient outcomes, particularly PFS.

## Basic framework and technical requirements in RANO 2.0

Since Levin proposed the first response assessment criteria in 1977 [35], several criteria have been introduced for evaluating treatment response in glioma clinical trials [36–41]. Until very recently, RANO-HGG (published in 2010) [37] and RANO-LGG (published in 2011) [38] had been widely used as response assessment criteria in numerous clinical trials [17, 33, 42]. Based on the accumulated knowledge and experience over the last decade since the publication of these two criteria, RANO 2.0 was introduced in 2023 [1]. These criteria have already been adopted in several imaging research studies [43, 44] and are expected to be implemented in many upcoming brain tumor trials. Therefore, radiologists need to become familiar with these criteria.

While RANO 2.0 inherits many elements from RANO-HGG and RANO-LGG, it indeed incorporates significant modifications (Table 1). Most notably, RANO 2.0 incorporates major revisions regarding the imaging modalities, target lesions, and timing used for response assessment. In this section, we will focus on these modifications, while addressing the remaining improvements in the latter part of this article.

**Table 1:** Comparison of radiographic response assessment between RANO-HGG, RANO-LGG and RANO 2.0

		RANO-HGG		RANO-LGG		RANO 2.0
Baseline scan for newly diagnosed glioma	Post-operation/pre-radiation		Post-operation/pre-radiation		Post-radiation	
Measurement technique	2D		2D or 3D (optional)		2D or 3D (optional)	
Tumor component evaluation						
Enhancing lesion	Required		Required		Required	
Non-enhancing lesion (i.e., T2/FLAIR lesion)	Required		Required		Omitted for glioblastoma; can be considered with agents that affect vascular permeability; required for non-enhancing or mixed tumor	
Confirmation scan (performed after > 4 weeks) for PD						
Within the first 12 weeks of completion of RT	Optional		Optional		Mandatory	
Beyond 12 weeks of RT					Optional; mandatory confirmation scan can be considered with therapy associated with high rates of pseudoprogression, or *IDH*-mt glioma	
Confirmation scan (performed after > 4 weeks) for CR/PR/MR	Mandatory		Mandatory		Mandatory	
Evaluation Glioma	High-grade glioma		Low-grade glioma		All glioma	

### Use of brain tumor imaging protocol

**Fig. 2:**
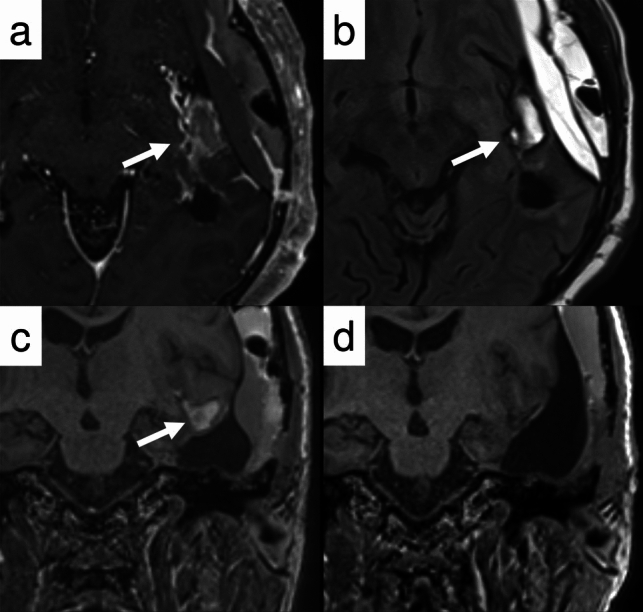
Postoperative imaging of a 72-year-old man with *IDH*-wildtype glioblastoma. **a** Contrast-enhanced T1WI shows a hyperintense lesion (arrow) adjacent to the resection cavity. **b** FLAIR sequence shows the same lesion as hyperintense with a smooth intersulcal contour. **c** Coronal T1WI confirms the lesion is confined between cerebral sulci. **d** Follow-up post-contrast T1WI shows complete resolution of the lesion, consistent with an intersulcal hematoma

Both in RANO (RANO-HGG and RANO-LGG) and RANO 2.0, the MRI sequences utilized for glioma response assessment includes non-contrast and contrast-enhanced T1WI, T2WI, and T2-FLAIR imaging. Careful evaluation of multiple sequences is essential both for assessing target lesions and for preventing the misdiagnosis of treatment-related changes (e.g., hemorrhage, infarction) as tumor progression (Fig. 2). While multiparametric evaluation methods incorporating advanced sequences such as diffusion-weighted image (DWI), magnetic resonance spectroscopy, and perfusion-weighted image (PWI), as well as positron emission tomography (PET) imaging with different tracers, are clinically significant [45–52], these modalities are not included in these criteria [53]. (Note: A separate RANO-PET framework for amino acid PET imaging was published in 2024 [54], but lies beyond the scope of this review.)

One of the major revisions in RANO 2.0 is the recommendation to use the Brain Tumor Imaging Protocol (BTIP) [55, 56], which was not addressed in the original RANO criteria that lacked protocols or sequence details. This standardized approach is strongly recommended to reduce imaging assessment variability across clinical sites. The BTIP is summarized in Table 2. Briefly, this protocol features parameter-matched pre and post-contrast 3D T1WI with high spatial resolution (≦ 1.5 mm isotropic), either 2D gapless (with ≦ 4 mm slice thickness) or 3D (≦ 1.5 mm isotropic) T2WI and T2-FLAIR images, and DWI. More advanced sequences, such as PWI, can be added to the BTIP protocol. For optimal consistency throughout the study period, participants should undergo MRI scans using either the same machine or equipment with equivalent magnetic field strength. While not mandatory, 3D T2WI and FLAIR sequences are included as options, enabling more precise volumetric measurements and offering advantages in the evaluation of *IDH*-mutant gliomas—typically non-enhancing lesions characterized by slow progression [57]. While both 2D and 3D measurements are acceptable, the measurement approach must be predefined in accordance with sponsor preferences to maintain methodological consistency throughout the study [2].

**Table 2:** Minimum standard 3 T MRI protocol

	3D-T1w Pre	Ax 2D FLAIR	3D FLAIR	Ax 2D DWI^a^	Ax 2D T2WI	3D-T1w Post
Sequence	IR-GRE	TSE/FSE	TSE/FSE	SS-EPI^b^	TSE/FSE	IR-GRE
Plane	Sagittal/axial	Axial	Sagittal/axial	Axial	Axial	Sagittal/axial
Mode	3D	2D	3D	2D	2D	3D
TR [ms]	2100	> 6000	90–140	> 5000	> 2500	2100
TE [ms]	Min	100–140	6000–10000	Min	80–120	Min
TI [ms]	1100	2000–2500^c^	2000–2500^c^	N/A	N/A	1100
Flip angle	10°–15°	90°/≧ 160°	Not specified	90°/180°		10°–15°
Frequency	≧ 172	≧ 256	≧ 244	≧ 128	≧ 256	≧ 172
Phase	≧ 172	≧ 256	≧ 244	≧ 128	≧ 256	≧ 172
NEX	≧ 1	≧ 1	Not specified	≧ 1	≧ 1	≧ 1
FOV	256 mm	240 mm	≦ 250 mm	240 mm	240 mm	256 mm
Slice Thickness	≦ 1.5 mm	≦ 4 mm	≦ 1.5 mm	≦ 4 mm	≦ 4 mm	≦ 1.5 mm
Gap/spacing	0	0	0	0	0	0
Parallel imaging	Up to 2×	Up to 2×	Up to 2×	Up to 2×	Up to 2×	Up to 2×

^a^b factor is 0, 500, 1000 s/mm^2^ ≧ 3 direction; it is acceptable to omit the intermediate shell b = 500 s/mm^2^, if needed

^b^In the event of significant patient motion and EPI is not an option, a radial acquisition scheme may be used

^c^Chosen based on vendor recommendation for optimized protocol and field strength

### Measurable lesion, non-measurable disease and target lesions

Similar to the original RANO-HGG/LGG criteria, RANO 2.0 initially classifies lesions as measurable or non-measurable, from which one or more target lesions are selected. Measurable disease is fundamentally defined as clearly demarcated lesions with a minimum short-axis diameter of 10 mm, extending across at least two slices in 2D imaging. For 2D assessment, select the imaging plane that displays the greatest extent of the lesion. In the context of volumetric analysis, target lesions are defined as those measuring at least 10 mm in each of the three orthogonal dimensions. For enhancing tumors, the enhancing component is measured, while for non-enhancing tumors, the T2 hyperintense lesion is measured. Non-measurable disease is those that do not meet the measurable lesion criteria, specifically lesions with a short-axis diameter less than 10 mm and/or ill-defined borders.

Special attention is required when measuring lesions with large cystic components, predominantly necrotic lesions, or lesions around the resection cavity [2]. For lesions predominantly composed of necrotic or cystic components, measurements should be confined to the solid portions of the tumor, excluding the dimensions of necrotic or cystic regions. When measuring lesions along the resection cavity, it is critical that the measurements exclude the cavity itself—the diameters should lie entirely within the lesion.

In cases presenting with multiple measurable lesions, target lesion selection should be limited to a minimum of two and a maximum of three lesions when assessing either enhancing or non-enhancing tumors. For tumors with both enhancing and non-enhancing components (namely mixed tumor presentation), target lesion selection may comprise up to two enhancing and two non-enhancing lesions. The primary criterion for target lesion selection should prioritize lesions demonstrating progressive enlargement, irrespective of their comparative dimensions. This selection principle applies to multiple lesion cases, where dimensional dominance is subordinate to documented growth progression in determining target status.

### Baseline scan

The immediate postoperative MRI scan, obtained within 48 h of surgery, has been used as the baseline MRI in most response criteria for newly diagnosed gliomas, including RANO-HGG [37] and immunotherapy RANO [39]. This approach was originally adopted to provide prompt assessment of residual tumor and establish an early reference point for tracking treatment effects. The surgical goal is typically to remove the enhancing portion of the tumor; however, nonspecific enhancement frequently develops in the wall of the surgical cavity within 48–72 h after surgery and can persist for weeks [58]. Therefore, RANO recommended obtaining baseline scans within 24–48 h after surgery to avoid misinterpreting these postoperative changes as residual enhancing disease.

By contrast, the modified RANO (mRANO) criteria recommend using the first post-radiotherapy MRI as the baseline for newly diagnosed gliomas to reduce the impact of the increased contrast enhancement from pseudoprogression (PsP) after CRT [40], and address the challenges associated with immediate postoperative scans including the presence of postoperative changes (blood products and edema) [59], and variability in corticosteroid dosing, timing of the scans, and imaging techniques used.

Recent research has shown that even with intraoperative MRI, postoperative changes related to surgery can cause contrast enhancement in a significant proportion of patients [60]. This finding, indicating that structural changes from surgery itself can lead to contrast enhancement in the immediate postoperative period, highlights a potential limitation of the original RANO approach. In contrast, mRANO aims to minimize the impact of radiotherapy and improve the accuracy of treatment response assessment by establishing the post-radiotherapy MRI as the baseline scan. RANO 2.0 also adopts this mRANO principle, setting the baseline scan at around 4 weeks (21–35 days) after the completion of radiation therapy, primarily to minimize the influence of PsP [1]. A recent retrospective large single-center study demonstrated that post-radiation scans showed a significantly higher correlation with PFS, a reliable endpoint in clinical trials, compared to immediate post-surgical scans, supporting the validity of this change in newly diagnosed or recurrent glioblastoma [61].

With the exception of glioblastoma, radiation therapy is not necessarily used as the initial treatment for glioma. Even in such cases, Ellingson et al. suggest that it is not necessary to use the scan immediately after surgery as the baseline [2]. Instead, it is preferable to take a pretreatment scan at a time when the effects of the surgical invasion have lessened, and within 14 days of the start of treatment. For recurrent gliomas, a pretreatment scan should also be used as baseline, with a standardized time interval (no more than 14 days) before the start of treatment, which is supported by recent evidence showing that recurrent glioblastoma can grow by an average of 15% over a 20-day period, with some tumors showing ≥ 25% growth even within 17 days—potentially compromising accurate response assessment [62].

## Response evaluation and interpretation in RANO 2.0

In addition to the fundamental modifications in the baseline conditions for response assessment, RANO 2.0 introduces substantial revisions to the response evaluation criteria themselves. Notable changes include the introduction of preliminary PD as a new assessment category with its associated confirmation scan protocol, as well as the exclusion of T2/FLAIR lesion evaluation from the assessment criteria for high-grade gliomas. This section describes the major modifications to response assessment categories in RANO 2.0.

### Basic assessment structure

Tumor response in the original RANO criteria for both high-grade gliomas and low-grade gliomas was primarily assessed based on changes in the Sum of the Products of Perpendicular Diameters (SPPD) of target lesion(s), measured from either the baseline or the nadir scan [37, 38]. The RANO-HGG response system comprised four categories—PD, stable disease (SD), partial response (PR), and complete response (CR)—whereas the RANO-LGG system included a fifth category: minor response (MR), allowing for more granular assessment in indolent tumors (Table 3). In both systems, confirmation scans were mandated for all response categories except PD, which permitted optional confirmation.

**Table 3:** Response categories in RANO 2.0

Category	CR	PR	MR	SD	PD^a^
Threshold to target lesion					
2D	No lesion	More than 50% reduction	Less than 50% but more than 25% reduction	Other than CR/PR/MR/PD	More than 25% increase
3D	No lesion	More than 65% reduction	Less than 65% but more than 40% reduction	Other than CR/PR/MR/PD	More than 40% increase
New measurable lesion	None	None	None	None	Any
Steroids	None	Stable or dose reduction	Stable or dose reduction	Stable or dose reduction	N/A
Clinical status	Stable or improved	Stable or improved	Stable or improved	Stable or improved	Deteriorated
Requirement	All	All	All	All	Any

*CR* complete response, *PR* partial response, *MR* minor response, *SD* stable disease, *PD* progressive disease

^a^PD can also be clearly determined by any of the following: (1) appearance of definite leptomeningeal disease, (2) clear progression of non-measurable lesions (increase in bidirectional diameters by at least 5 × 5 mm to > 10 × 10 mm), or (3) unequivocal progression of existing non-target lesions (requiring ≥ 25% increase in sum of products of perpendicular diameters or ≥ 40% increase in volume)

The foundational framework and structure of these systems is largely preserved in RANO 2.0, although several modifications were introduced. Specifically, the updated criteria apply RANO-HGG principles to enhancing lesions and RANO-LGG principles to non-enhancing lesions. While this structural consistency is maintained, some conceptual elements have been substantially revised. In the following subsections, we address two major modifications related to treatment response evaluation introduced in RANO 2.0:

### Evolution of progressive disease assessment

The determination of PD has undergone significant modification in RANO 2.0, primarily due to the challenges posed by PsP. PsP, occurring in up to 40% of cases within 12 weeks post-treatment [63–66], presents as temporary enhancement that reflects treatment-induced inflammation rather than disease progression. RANO-HGG criteria addressed this by not classifying new enhancement within the radiation field as PD during the initial 12 weeks post-treatment [37]. While this aimed to prevent premature treatment discontinuation due to misinterpretation of PsP as PD, the approach complicated accurate PFS measurement in cases of early recurrence. The complexity was further amplified by RANO criteria’s optional requirement for confirmation scans when evaluating disease progression, thereby introducing additional uncertainty into the progression assessment process.

To address these challenges, RANO 2.0 has implemented the following key changes to PD assessment [2, 67, 68].

- Implementation of preliminary PD, defined by either a more than 25% increase in SPPD or new enhancing lesions during 12 weeks initial treatment.
- Mandatory confirmation scans after 4 or 8 weeks for new enhancement within 12 weeks post-treatment if the patient is clinically stable.
- If the subsequent scan confirmed progression, the date of progression should be backdated to the time initial tumor progression was noted.
- If enhancing lesions emerge after the initial 12 weeks period, PD will be determined without a confirmatory scan unless specifically required by the clinical trial sponsor.
- Optional confirmation scans are warranted for new enhancement detected outside the radiation field or beyond 12 weeks post-treatment in the context of immunotherapy, given the recognized high incidence of potential immunotherapy-associated PsP [39, 69].

These changes enable earlier differentiation between PsP and true PD while facilitating more accurate PFS measurement [61].

### Elimination of T2/FLAIR lesion from enhancing tumor assessment

Pseudoresponse and non-enhancing tumor progression represent two distinct radiological phenomena in glioma patients treated with anti-angiogenic therapy (particularly bevacizumab). Pseudoresponse is characterized by rapid reduction in contrast enhancement that may not reflect true tumor response, as it primarily results from decreased vascular permeability rather than actual tumor reduction [70]. In contrast, non-enhancing tumor progression manifests as expanding T2/FLAIR abnormality despite stable or decreased contrast enhancement, indicating infiltrative tumor growth [71].

In response to these complex imaging findings, the RANO criteria have emphasized the importance of evaluating both contrast-enhanced T1WI and T2/FLAIR sequences. While T2/FLAIR evaluation is valuable for detecting pseudoresponse and identifying non-enhancing tumor progression, there remains a risk of misinterpreting treatment-related T2/FLAIR signal changes as progressive disease. Furthermore, non-enhancing tumor progression typically transitions to enhancing progression within 1–2 months, and notably, previous studies have failed to demonstrate significant correlations between non-enhancing lesion measurements and either PFS or survival outcomes [61, 72, 73].

RANO 2.0 has modified the relative weight given to T2/FLAIR imaging in glioblastoma assessment, placing greater emphasis on contrast-enhancing lesions. The modification reflects dual considerations: firstly, the limited prognostic utility of non-enhancing lesion assessment in glioblastoma, and secondly, the operational burden of complex volumetric analyses. Moreover, the omission of T2/FLAIR evaluation criteria for enhancing tumors in RANO 2.0 may mitigate the potential misclassification of transient post-treatment change as PD [74]. Nevertheless, the criteria maintain that T2/FLAIR hyperintensity should be incorporated into response evaluation during anti-angiogenic therapy, analogous to the approach used in predominantly non-enhancing tumors. This specific recommendation persists because anti-angiogenic agents can mask tumor progression through vascular normalization while infiltrative growth continues beyond contrast-enhancing regions.

## Practical application of RANO 2.0: representative clinical scenarios

As with the major modifications discussed above, a notable feature of RANO 2.0 is the adoption of three imaging-based categories: enhancing tumor, non-enhancing tumor, and mixed tumor, which is defined by the concurrent presence of both enhancing and non-enhancing tumor components. The major revisions outlined in preceding sections and these newly introduced imaging categories are intricately interrelated, with varying weights and frequencies depending on the clinical contexts, making uniform interpretation challenging. To facilitate clearer understanding of these concepts, the following section presents representative cases illustrating how RANO 2.0 principles can be applied in dynamic clinical scenarios. While that treatment response criteria are primarily intended for clinical trials [75–77], the types of disease progression and treatment response observed in such settings are often analogous to those encountered in routine imaging assessments. Accordingly, we interpret the following cases through the lens of RANO 2.0 to help the reader become more familiar with the updated structure and conceptual framework of RANO 2.0 through its practical application.

*Case 1 newly diagnosed, no residual enhancing tumor, new measurable lesion after 12 weeks of CRT (PD)*

**Fig. 3:**
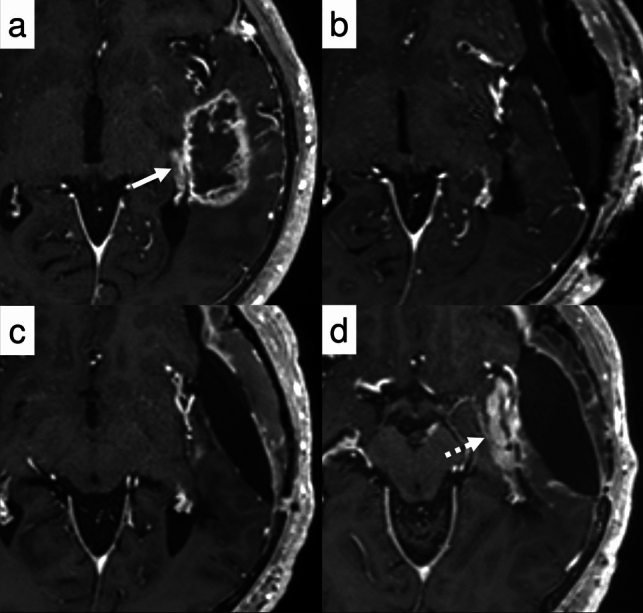
Case 1: A 72-year-old man with *IDH*-wildtype glioblastoma presented with a contrast-enhancing tumor (**a**, arrow), which was completely resected (**b**). He subsequently underwent chemoradiotherapy (CRT). Four weeks after completing CRT—corresponding to the RANO 2.0 baseline scan—no residual tumor was observed on contrast-enhanced T1WI (**c**). A new measurable lesion (12 × 17 mm, dotted arrow) was detected 4 months later (**d**), fulfilling the criteria for progressive disease

Glioblastoma treatment follows a multimodal approach [78]. Case 1 (Fig. 3) demonstrates a typical treatment course for this malignancy. Most histologically classified high-grade gliomas, with glioblastoma being a prime example, are categorized as enhancing tumors according to RANO 2.0. The standard protocol involves maximal safe surgical resection of the contrast-enhancing lesion, followed by radiation therapy delivered to the tumor volume with an appropriate margin.

As seen in Case 1, where complete removal of the enhancing tumor was accomplished, evaluating the treatment response presents a specific challenge. In situations where there is no measurable disease on the initial imaging, stable disease (SD) represents the best possible outcome during follow-up; without a measurable target lesion at the start, objective responses (MR/PR/CR) cannot be achieved. The assessment remains SD unless either new measurable disease develops or a pre-existing non-measurable component (limited to contrast-enhancing components in contrast-enhancing tumors) shows sufficient growth (reaching ≥ 5 × 5 mm to ≥ 10 × 10 mm) compared to initial size, in which case the response is classified as PD.

Under RANO 2.0, PD can be determined when new measurable enhancing lesions appear more than 12 weeks after treatment completion (Fig. 3), though specific clinical trial protocols may necessitate confirmation scans.

*Case 2 newly diagnosed, non-enhancing residual tumor, new measurable lesion within 12 weeks of CRT (PsP)*

**Fig. 4:**
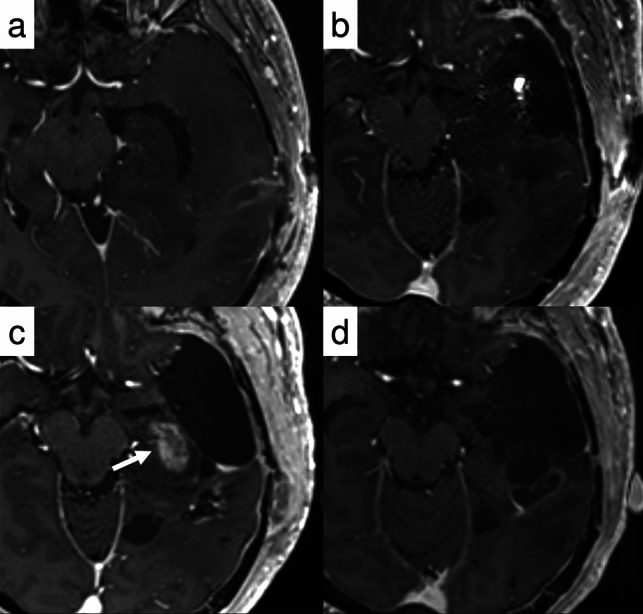
Case 2: A 37-year-old man presented with a non-enhancing tumor in the left temporal lobe (**a**). Maximal safe resection was performed, and histopathology confirmed an *IDH*-mutant astrocytoma, WHO grade 3 (**b**). The patient underwent concurrent chemoradiotherapy (CRT). One month after completing CRT, baseline post-treatment imaging showed nodular contrast enhancement (arrow) along the margin of the resection cavity (**c**). The first confirmation scan (not shown) demonstrated spontaneous regression of the enhancement, and complete resolution was observed on the 3-month follow-up (**d**). Based on this clinical and radiological course, a diagnosis of pseudoprogression (PsP) was made

Case 2 (Fig. 4) exemplifies typical PsP occurring within 12 weeks following completion of concurrent CRT, without accompanying symptoms. For such cases, while both criteria essentially avoid designating these changes as PD, their approaches differ: the original RANO criteria exclude new enhancement within three months post-treatment from PD assessment, whereas RANO 2.0 requires confirmatory scans to actively verify the absence of true progression.

Notably, in *IDH*-mutant cases, it is important to remember that PsP may occur beyond 12 weeks post-CRT [79–82]. In recognition of this characteristic, RANO 2.0 suggests that confirmation scans may be considered for new enhancement appearing after 3 months post-treatment in *IDH*-mutant cases.

*Case 3 newly diagnosed, enhancing or mixed residual tumor, new measurable lesion after 12 weeks of CRT (PD)*

**Fig. 5:**
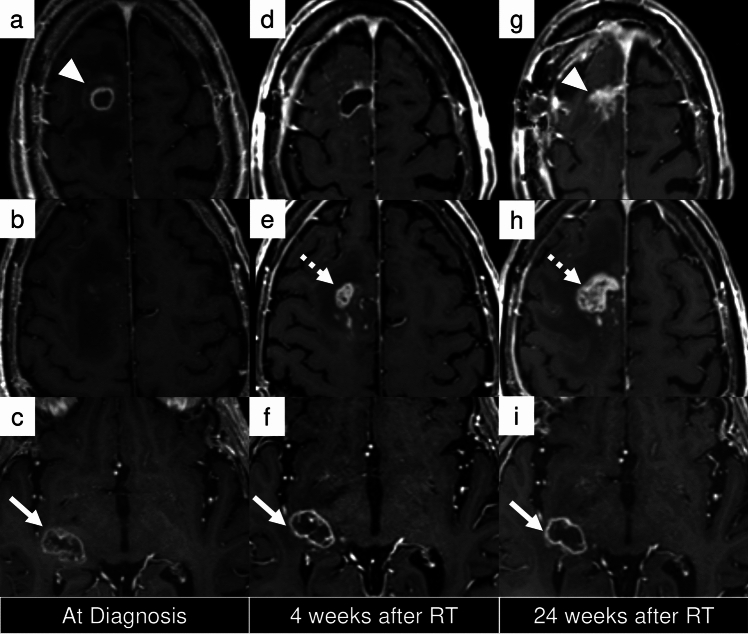
Case 3: A 79-year-old man with glioblastoma, *IDH*-wildtype presented with multiple enhancing and non-enhancing lesions, including a dominant right frontal lesion (**a**, arrowhead). Partial resection of the right frontal lesion was performed (**d**), and concurrent chemoradiotherapy (CRT) was administered, including coverage of a non-resected right temporal lesion (**c**, arrow). At 4 weeks post-CRT, new non-measurable enhancing lesions appeared around the resection cavity (**e**, dashed arrow), and remained stable through 12 weeks post-CRT (not shown), without evidence of progression. At 24 weeks (**h**), the lesions (dashed arrow) had enlarged and met the criteria for progressive disease (PD). Irregular contrast enhancement was also noted around the surgical cavity (**g**, arrowhead). In contrast, the right temporal lesion, designated as a target lesion, showed no measurable change over time (**f, i**, arrows). Panels **a–c**, **d–f**, and **g–i** represent equivalent axial, sagittal, and coronal levels, respectively, at diagnosis, early, and late follow-up

While complete resection is optimal for glioma treatment, cases involving multiple lesions, as in case 3 (Fig. 5), often present challenges that preclude total removal, even with advanced techniques such as awake surgery. According to RANO 2.0, beyond 3 months after completion of radiotherapy, PD can be determined when pre-existing measurable lesions demonstrate either a ≥ 25% increase in 2D measurements or a ≥ 40% increase in 3D volume. However, some clinical trials may require confirmation scans, in which case these findings are initially classified as preliminary PD. In such trials, treatment continues while awaiting a confirmation scan performed at least 4 weeks later. PD is confirmed if there is a further 25% increase in SPPD or 40% increase in volume, with the date of progression backdated to the preliminary PD date. If these criteria are not met, the case is classified as PsP, and the clinical trial continues. Although the emergence of new measurable lesions in this case was sufficient to establish PD, it is important to note that PD assessment in cases with multiple measurable lesions generally requires calculation of the sum of SPPDs (in 2D imaging) or volumetric measurements (in 3D imaging). This principle is especially relevant when evaluating progression of non-target lesions, which requires ≥ 25% increase in sum of products of perpendicular diameters or ≥ 40% increase in volume of the lesion(s), with these measurements being added to the sum of target lesions to determine overall disease status.

*Case 4 newly diagnosed, residual enhancing tumor, new measurable lesion within 12 weeks of CRT (PD)*

**Fig. 6:**
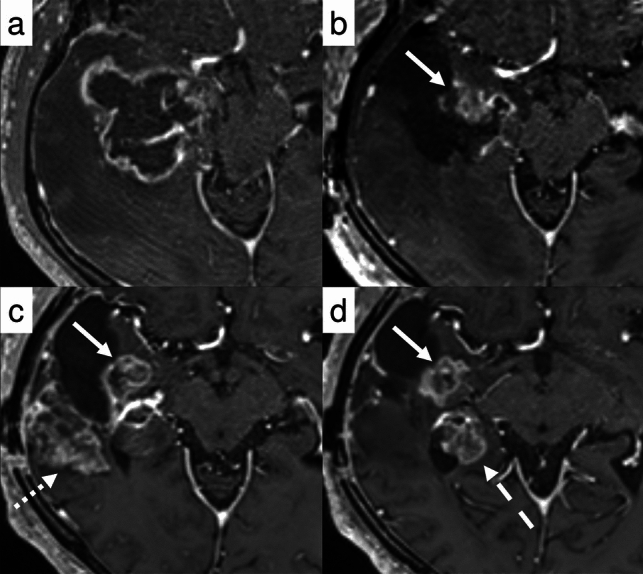
Case 4: A 73-year-old man with right temporal glioblastoma, *IDH*-wildtype, underwent maximal safe resection followed by adjuvant chemoradiotherapy (CRT). Postoperative contrast-enhanced T1WI demonstrated a small residual lesion in the medial margin of the surgical cavity (**b**, solid arrow), which remained stable in size throughout the observation period (**c, d**). At 8 weeks following CRT completion, a new contrast-enhancing lesion emerged along the posterior resection margin (**c**, dotted arrow), meeting RANO 2.0 for preliminary progressive disease (preliminary PD). However, this lesion spontaneously resolved on the 12-week follow-up scan (**d**), supporting a diagnosis of PsP. Concurrently, a new contrast-enhancing lesion appeared in the right hippocampus (d, dashed arrow). Progressive disease was subsequently confirmed based on follow-up imaging performed 4 months after treatment completion (not shown)

Case 4 (Fig. 6) warrants systematic evaluation following RANO 2.0, as it presents a complex clinical course with multiple findings requiring careful interpretation. First, there is a measurable lesion in the medial temporal lobe that maintains SD. A contrast-enhancing lesion dorsal to the resection cavity transiently enlarged within 3 months after CRT, but subsequently decreased in size, consistent with PsP. However, the appearance of new measurable lesions beyond the 3-month window led to an overall assessment of PD[68].

*Case 5 newly diagnosed, non-enhancing tumor (MR)*

In RANO 2.0, non-enhancing tumors typically include lower-grade *IDH*-mutant gliomas, though on rare occasions, *IDH*-wildtype gliomas may also fall into this category when they lack contrast enhancement. For these tumors that present without measurable contrast-enhancing lesions, measurable disease is defined as a clearly demarcated T2/FLAIR hyperintense lesion with a minimum short-axis diameter of 10 mm.Unlike their *IDH*-wildtype counterparts, *IDH*-mutant low-grade gliomas are characterized by slow growth and gradual treatment responses in both clinical trials and routine practice. To better capture modest but meaningful responses, RANO-LGG introduced a “minor response” category, which RANO 2.0 has retained for non-enhancing tumors [57, 83].

**Fig. 7:**
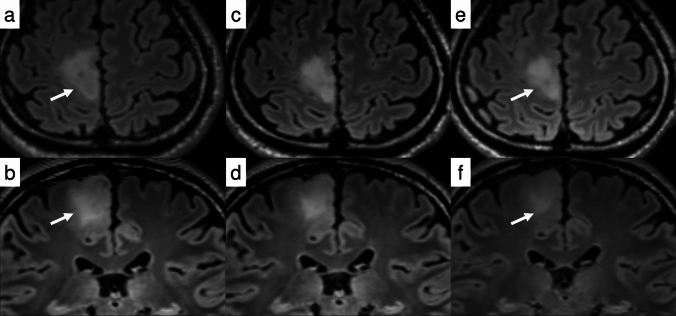
Case 5: A man in his 20 s presented with a right frontal lobe tumor. Biopsy revealed an *IDH*-mutant diffuse glioma, histologically classified as WHO Grade 2 (**a, b**). The tumor gradually enlarged during follow-up, and the patient was subsequently enrolled in a clinical trial involving a novel therapeutic agent. After initiation of treatment, the lesion demonstrated a slow but continuous reduction in size over time (**c, d**: 1 year after treatment initiation; **e, f**: 2 years after treatment initiation). All images are 3D FLAIR sequences. The top row shows axial reconstructions; the bottom row shows coronal reconstructions. Arrows indicate the lesion of interest at the initial and latest timepoints

Treatment response must be evaluated in comparison to pretreatment baseline scans, with particular attention to temporal changes, as lesion reduction typically occurs gradually over years, as demonstrated in case 5 (Fig. 7). Ellingson et al. addressed this challenge of temporal assessment through two innovative approaches. First, they identified “yo-yoing”—inconsistency in measurements—as a significant limitation of 2D assessment, advocating for volumetric measurement using 3D scans [57]. Additionally, they proposed the “digital flipbook” method to facilitate visual detection of temporal changes in imaging [84]. While radiologists have traditionally performed such temporal comparisons, implementation of these advanced tools in clinical viewers could enhance detection of subtle changes [85, 86]. However, given the current challenges in establishing standardized segmentation workflows and the associated labor intensity, RANO 2.0 maintains volumetric analysis as an optional approach.

*Case 6 recurrent, residual non-measurable enhancing tumor (pseudoresponse and non-enhancing tumor progression)*

**Fig. 8:**
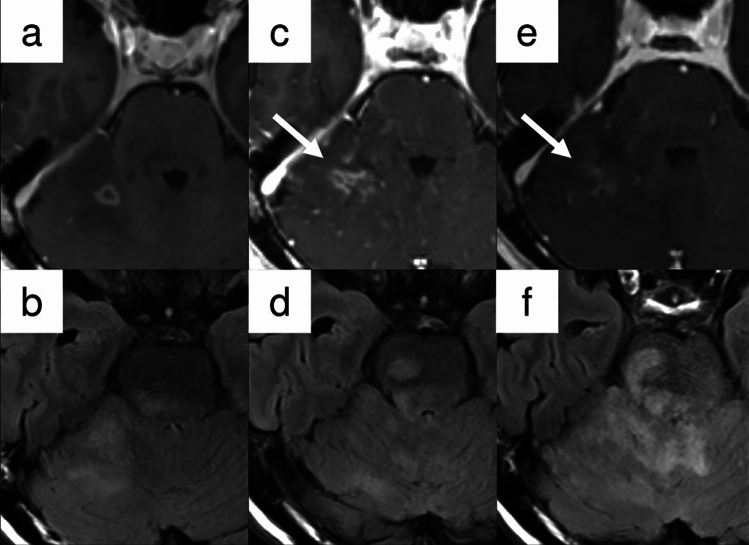
Case 6: A 75-year-old man presented with a tumor involving the cerebellum and pons. Stereotactic biopsy suggested diffuse midline glioma with H3K27-altered status (**a** and **b**). Eight months following completion of concurrent CRT, surveillance imaging revealed increased contrast-enhancing foci (arrow), raising concern for disease progression (**c** and **d**). The patient was subsequently initiated on second-line chemotherapy including bevacizumab. Follow-up imaging at one month demonstrated rapid resolution of contrast enhancement (arrow) (**e**) as well as progression of T2-FLAIR lesion (**f**); consistent with pseudoresponse and non-enhancing tumor progression, a characteristic imaging pattern associated with anti-angiogenic therapy

Anti-angiogenic therapy such as bevacizumab can cause reduction in enhancement within 1–2 days after administration, with a radiographic response in 25–60% of patients. As previously mentioned, RANO 2.0 excludes T2/FLAIR assessment in treatment response evaluation for enhancing tumors. Case 6 (Fig. 8) exemplifies why modified response criteria may be necessary in clinical trials of anti-angiogenic agents, as conventional contrast enhancement patterns can be misleading and may not accurately reflect actual disease status.

While this article does not detail RANO 2.0 regarding steroid use and clinical deterioration, it is important to note that some cases with diffusely infiltrative patterns may be classified as PD based on clinical deterioration despite minimal imaging changes. This underscores that radiologists, like their colleagues in neuro-oncology, should remain mindful that imaging findings, while providing objective metrics, may not fully capture the complete spectrum of disease progression.

*Case 7 recurrent, residual mixed tumor, with measurable enhancing lesion (PD)*

*Case 8 recurrent, residual mixed tumor, with non-measurable enhancing lesion (SD)*

As previously mentioned, RANO 2.0 has adopted an “imaging-based” classification system that categorizes gliomas into enhancing tumors, non-enhancing tumors, and mixed tumors, implementing a unified response assessment approach. Regarding enhancing and non-enhancing tumors, this classification system parallels the original RANO criteria, which employed two distinct assessment systems based on histological classification (high-grade versus low-grade), as in practice, enhancing tumors typically correspond to glioblastoma, *IDH*-wildtype, while non-enhancing tumors generally align with *IDH1/2*-mutant low-grade gliomas [87, 88].

The newly introduced category of mixed tumors appears less intuitive and is characterized by measurable contrast-enhancing lesions within relatively extensive non-enhancing legions. This category appears to correspond to an imaging pattern historically described as secondary glioblastoma or gliomatosis cerebri (with enhancing foci), though it should be noted that these historical terms are no longer recommended under current molecular-based classification schemes.

**Fig. 9:**
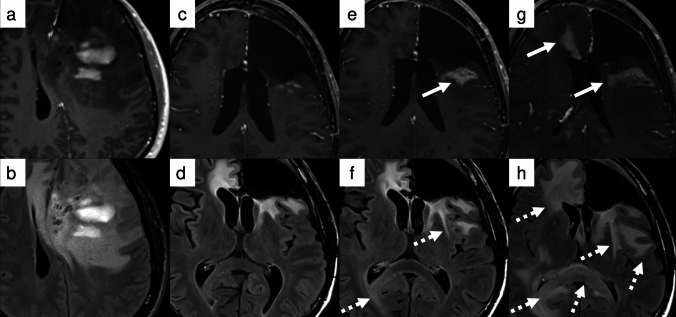
Case 7: A 33-year-old male presented with a large left frontal lobe tumor demonstrating focal enhancement (**a** and **b**). Surgical resection was performed, and histopathological analysis revealed WHO grade 3 oligodendroglioma (**c** and **d**). The patient received adjuvant chemoradiotherapy. At 18 months post-treatment, surveillance imaging demonstrated enlargement of residual enhancement focus (arrow, **e**) concurrent with equivocal non-enhancing progression (dotted arrow, **f**). Based on these radiological findings, progressive disease was diagnosed. Despite initiation of salvage chemotherapy, both the enhancing (arrow, **g**) and non-enhancing components (dotted arrow, **h**) showed continued progression

**Fig. 10:**
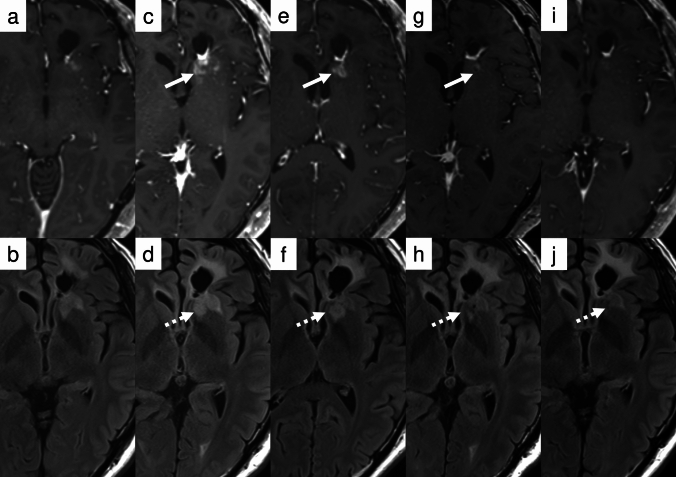
Case 8: A 35-year-old male underwent biopsy that revealed a histologically low-grade glioma. Molecular biological examination identified *IDH*-wildtype status with *TERT* promoter and *BRAF* V600E mutations (**a** and** b**). Despite these molecular findings, the patient was initially placed under careful observation. At 27 months after biopsy, newly developed enhancing foci appeared, raising concern for tumor progression (arrow, **c**). Targeted combination therapy with dabrafenib and trametinib was initiated. Follow-up scans at 3 months (**e, f**), 7 months (**g, h**), and 9 months (**i, j**) demonstrated near-complete disappearance of the enhancing lesions (white arrows, **c** and **e**) and gradual reduction of the non-enhancing T2/FLAIR abnormalities (dotted arrows, **d, f, h, j**). Although both components showed radiographic improvement, they were classified as non-measurable under RANO 2.0. The response was thus formally categorized as stable disease, though the clinical and radiologic course was consistent with a minor response or near-partial response

The imaging pattern characteristic of mixed tumors may be observed in several tumor types described in WHO CNS5:

- *IDH*-mutant high-grade glioma (Fig. 9) [89, 90].
- Certain *IDH*-wildtype tumors (Fig. 10), predominantly those diagnosed as molecular glioblastoma [20, 91, 92].
- Some pediatric-type high-grade gliomas, including those with H3K27a and H3G34m [93–95].

Three important caveats should be noted regarding this classification: First, while these tumor types may present as mixed tumors, their classification should be determined on a case-by-case basis, as these categorical distinctions may be significantly influenced by clinical trial design considerations. Second, while most histological glioblastomas also present with T2 hyperintense lesions accompanying contrast enhancement, cases where contrast-enhancing lesions are considered representative of disease activity (i.e., cases with relatively limited non-enhancing disease) should be classified as enhancing tumors. Notably, there are no clear definitions regarding what proportion of total tumor volume should be considered “representative” enhancing disease, suggesting this threshold may need to be specified in individual trial protocols. Lastly, the mixed tumors are relatively rare entities. For instance, *IDH*-mutant, WHO grade 4 gliomas constitute merely 2% of all gliomas [96], and H3-altered tumors occur at similarly low frequencies [95]. Furthermore, considering that maximal safe resection—whether of contrast-enhancing lesions with or without FLAIR-evident abnormalities (supramarginal resection [97–99])—represents the standard of care, there are relatively few cases that warrant monitoring as mixed tumor components following appropriate surgical intervention. Although this perspective may include some subjective assessment, it suggests that the primary candidates for such monitoring might be limited to cases where complete resection is deemed challenging, such as tumors involving multiple lobes with unresectable enhancing components, or recurrent *IDH*-mutant gliomas.

In the assessment of mixed tumors, up to two target lesions can be designated for both enhancing and non-enhancing components, as noted previously. These components must be monitored separately with dedicated target lesions. The overall response classification follows specific criteria (Table 4): PD is determined if either component demonstrates PD; when one component shows MR/PR/CR while the other shows SD, the overall response is classified as MR/PR. A CR classification requires both components to demonstrate CR, and similarly, SD is only assigned when both components exhibit SD. Importantly, the assessment of non-enhancing components should exclude vasogenic edema, though this distinction becomes particularly challenging in cases with significant blood–brain barrier disruption, despite being relatively straightforward in typical mixed tumors.

**Table 4:** Response category when mixed glioma is treated

	Enhancing lesion	Non-enhancing lesion	Overall response
Response category	PD	Any	PD
	Any	PD	
	SD	SD	SD
	PR	SD	PR^a^/MR
	SD	PR/MR^b^	
	PR	PR/MR^b^	
	CR	CR	CR

^a^If PR is determined based on reduction in tumor size of enhancing disease, non-enhancing disease must be at least stable and vice versa

^b^MR applies to non-enhancing disease: MR can only be determined if the enhancing disease is at least stable

## Future perspectives

Where will RANO go from here? Just as the decade between RANO and RANO 2.0 brought significant knowledge and advancements, will RANO 2.0 continue to evolve through upcoming clinical trials and the introduction of novel therapeutic agents?

It should be noted that this past decade has also witnessed the widespread introduction of artificial intelligence (AI) into clinical medicine, fundamentally transforming the landscape of medical practice [100–106]. Within this context, the AI-RANO initiative represents a pivotal development, aiming to standardize and clinically implement AI technologies in brain tumor management [107, 108]. The convergence of clinical insights with advanced AI capabilities promises to enhance the objectivity and precision of response assessment through automated tumor segmentation [109], volumetric analysis [110], and identification of subtle imaging biomarkers that may escape conventional evaluation [111].

As we stand at this crossroads of accumulated clinical experience and emerging AI technologies, the future evolution of response assessment criteria in neuro-oncology will likely be shaped by both the lessons learned from novel therapeutic approaches and the transformative potential of artificial intelligence-driven methodologies. Throughout this evolution, we fervently hope that radiologists will remain at the forefront, guiding and interpreting these advancements.

## Conclusions

RANO 2.0 represents the most current response assessment criteria for gliomas. While designed primarily for clinical trials—aiming to standardize imaging interpretation and optimize PFS measurements to better correlate with patients’ overall survival—strict application of these criteria may present challenges in routine clinical practice.

However, as radiologists and members of the neuro-oncology team involved in brain tumor patient care, understanding these assessment principles is crucial, even when modifications may be necessary for day-to-day practice. This understanding enhances our ability to contribute meaningfully to treatment monitoring and decision-making.
